# Specificity of CD200/CD200R pathway in LPS-induced lung inflammation

**DOI:** 10.3389/fimmu.2022.1092126

**Published:** 2022-12-15

**Authors:** Dany Patoine, Karine Bouchard, Anne-Marie Lemay, Elyse Y. Bissonnette, Jean-Francois Lauzon-Joset

**Affiliations:** ^1^ Centre de Recherche de l'Institut Universitaire de Cardiologie et de Pneumologie de Québec - Université Laval, Québec, QC, Canada; ^2^ Department of Medicine, Faculty of Medicine, Université Laval, Quebec, QC, Canada

**Keywords:** Acute respiratory distress syndrome (ARDS), alveolar macrophages (AM), lung homeostasis, MAP kinase, broncho alveolar lavage (BAL), mucosal immunity, anti-infl ammatory, immune check point

## Abstract

**Introduction:**

At lung mucosal surfaces, immune cells must initiate inflammatory response against pathogen without inducing tissue damage. Loss of this equilibrium can lead to acute respiratory distress syndrome (ARDS), a severe lung inflammatory disease characterized by excessive inflammation and dysregulation of anti-inflammatory pathways.

**Methods:**

To investigate the role of anti-inflammatory pathway CD200/CD200R in lung inflammatory response, we administered LPS intratracheally in CD200 KO and wild type (WT) rats. Inflammation was evaluated using bronchoalveolar lavage (BAL) cellularity. Lung injury was measured by total protein level in BAL fluid, and levels of proinflammatory cytokines (TNF, IL-6) and chemokines (CXCL2, CCL2) were determined in BAL supernatants. In a second series of experiments, recombinant CD200Fc was administered to KO rats to restore the anti-inflammatory response.

**Results:**

At baseline, CD200 KO rats did not show sign of inflammation, however KO rats had lower number of alveolar macrophages. In addition, LPS administration induced greater pulmonary edema in CD200 KO rats. This was accompanied with a higher recruitment of neutrophils as well as levels of TNF, IL-6, CXCL2, and CCL2 in BAL compared to WT rats. CD200Fc administration in KO rats reduced neutrophil accumulation and TNF and CXCL2 levels in BAL. Interestingly, the increased inflammatory response of CD200 KO rats could be attributed to greater activation potential of alveolar macrophages with higher levels of ERK and P-ERK MAPK.

**Conclusion:**

This study shows that lung inflammatory response is exacerbated in absence of CD200 in an experimental model of ARDS in rats. In addition, CD200/CD200R pathway shows selective regulation of acute lung inflammation and cannot completely abrogate the complex LPS-induced inflammatory response. However, addition of CD200 agonist in a multi-target therapy strategy could have beneficial impacts.

## Introduction

Acute respiratory distress syndrome (ARDS) is a severe inflammatory lung disease caused by pulmonary insults and indirect systemic inflammatory responses (1). Recently, ARDS was identified as the common complication of SARS-CoV-2 infection (2). ARDS is characterized by influx of inflammatory cells such as neutrophils and recruited macrophages, acute diffuse alveolar damage, and protein rich exudates in alveolar spaces (3). Lung macrophages play a key role in ARDS pathogenesis (4, 5) as they are major players in both initiation and resolution of lung inflammation (6). Furthermore, anti-inflammatory pathways play an important role to control the severity of ARDS. Emerging evidence suggests that early inflammatory events initiate also resolution of inflammation which is crucial to return to homeostasis (7). The pro-resolution strategies are multiple and depend on the stimuli and tissues affected (8). This process involves endogenous anti-inflammatory mediators and expression of co-inhibitory surface molecules including CD200-CD200R (8–10).

CD200 is a highly conserved transmembrane glycoprotein expressed on a variety of cells including macrophages, dendritic cells, T cells, B cells, endothelial cells, and epithelial cells (11–13). CD200 does not induce signaling in cell expressing it, given its short cytoplasmic tail (14). To induce anti-inflammatory signaling, CD200 must bind to its receptor CD200R which is expressed on lymphoid and myeloid cell lineages only (14, 15). Activation of CD200R induces immune suppression in different models of inflammation including arthritis, transplantation, and neuroinflammation (16). Faster and more pronounced inflammatory responses are observed in absence of CD200 in model of experimental autoimmune encephalomyelitis and an increased susceptibility to collagen-induced arthritis (17), whereas treatment with recombinant CD200 reduces disease severity in both models (18, 19).

Interestingly, in the lung, CD200R is highly express by alveolar macrophages (AMs) (12), supporting an important role of these cells in controlling inflammation and maintaining lung homeostasis (20). However, fewer studies have investigated the role of CD200/CD200R pathway on lung immune response. Snelgrove et al. showed that mice lacking CD200 have an amplified inflammatory response to influenza virus infection which help to eliminate the virus but, lack of inflammatory control by CD200 causes a more severe lung damage compared with wild type (WT) mice (21). In an animal model of acute allergic asthma, addition of CD200R ligand intratracheally before allergen challenge reduces airway hyperresponsiveness, IL-13 levels in bronchoalveolar lavage (BAL), and lung dendritic cell accumulation, without reducing eosinophil recruitment (22, 23). These studies show the fine balance in CD200 role to reduce inflammatory responses, while maintaining adequate pathogen surveillance in the lungs.

Given the intricacy of CD200/CD200R pathway on controlling immune responses depends on stimuli and tissues, we investigated the role of CD200 in a model of acute respiratory distress syndrome (ARDS) induced by LPS using CD200 knockout (KO) Sprague Dawley rats. We show that lack of CD200 causes a remarkable reduction of AM number in BALs of naïve rats and more important lung inflammation and injury after LPS administration. We also demonstrate that without the inhibitory signal of CD200, AMs have a higher potential to initiate an inflammatory cascade.

## Material and methods

### CD200 KO rat development

Sprague-Dawley CD200 KO rat strain, namely Crl : CD(SD)-CD200^em1EB^, was developed by the Transgenesis and animal modelling platform (CRCHUM, Montréal, QC, Canada) using CRISPR/Cas9 technology. A deletion of 4 nucleotides in CD200 exon 3 that results in a premature stop codon was confirmed by DNA sequencing (**Supplementary Figure 1A**). Protein deficiency was verified by flow cytometry on total white blood cells using PE anti-rat CD200 antibody (clone OX-2, Biolegend, San Diego, CA) (**Supplementary Figure 1B**).

### Animals and LPS administration

CD200 KO rats were bred and maintained at the Centre de Recherche de l’Institut Universitaire de Cardiologie et de Pneumologie de Québec (Quebec City, QC, Canada). Control rats CD (SD) were obtained from Charles-River Laboratories (Saint-Constant, QC, Canada). The protocol was approved by Laval University Animal Care Committee in accordance with the guidelines of the Canadian Council on Animal Care. Male rats 7-8-weeks-old received intratracheal administration of 0.5 µg LPS (E.coli, O55:B5; Millpore-Sigma, St. Louis, MO) in saline solution. In a second set of experiments, 0.02 nmol of either recombinant mouse CD200 fused with an IgG1 Fc (CD200Fc; R&D Systems, Minneapolis, MN) or recombinant mouse IgG1 Fc (Sham; R&D Systems) was administrated with LPS to KO rats. Animals were anesthetized with ketamine/xylazine and exsanguinated *via* abdominal aorta at different times after LPS administration.

### Measurement of airway inflammation

BAL was performed as previously described (24) with phosphate-buffered saline (PBS)-EDTA 10 mM. First 5 mL of BAL was kept for cytokines/chemokines measurement. Enzyme-linked immunosorbent assay (ELISA) were performed using DuoSet^®^ kits (R&D System) to measure TNF, IL-6, CCL2, and CXCL2 according to the manufacturer’s instructions. Total proteins in BAL was measured with the DC protein assay kit (Bio Rad, Mississauga, ON, Canada). Cells for differential counts were identified by flow cytometry (**Supplementary Figure 2**).

### Flow cytometry

Following cell count, 1 × 10^6^ cells from BAL were stained with the following panel: MHC-Class II-PerCP (BD Biosciences, Mississauga, Ont, Canada), CD11b-APC (BD), CD3-BV605 (BD), CD45R-BV786 (BD), CD172-AF700 (Bio-Techne, Toronto, Ont, Canada), CD43-AF405 (Bio-Techne), CD200-PE (BioLegend, San Diego, CA), CD200R1-Biotin (Biolegend) followed by Strep-APC-eFluor780 (Life Technologies, Mississauga, Ont, Canada).

For MAPK experiments, cells were fixed and permeabilized with Intracellular Flow Cytometry Kit (Cell signaling technology, Whitby, Ont, Canada) according to the manufacturer’s instructions and stained with P-p44/42-Erk1/2-PE, p44/42-ERK1/2-AF647, P-p38-AF647, p38-PE (all from Cell signaling). Flow cytometry acquisition was performed on a LSR Fortessa (BD) and analysed with FlowJo 10.8 (FlowJow, Ashland, OR).

### Statistical analysis

6-8 rats were used per group in two independent experiments for each analysis. Prism 9.4.1 (GraphPad Software, La Jolla, CA) was used for all statistical analyses, which included either Student’s t test, Mann-Whitney, ANOVA followed by Fisher’s LSD test or the Kruskal-Wallis followed by Dunn’s uncorrected post-test. Data are presented as mean ± SEM, and P values less than 0.05 were considered significant.

## Results

**Figure 1:**
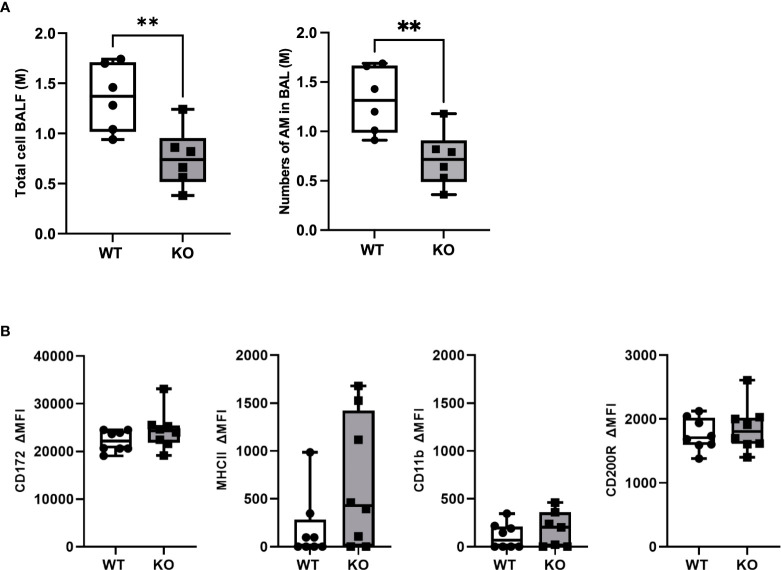
CD200 KO rats do not display signs of lung inflammation at baseline, but have reduced number of alveolar macrophages. Bronchoalveolar lavages (BAL) were performed on CD200 KO and WT rats at baseline. **(A)** Total BAL cells were counted after trypan blue staining (left panel) and numbers of AM were confirmed by flow cytometry analysis (right panel). **(B)** Expression of activation marker on alveolar macrophages was assessed by flow cytometry using delta mean fluorescence intensity. Data are from individual rats and displayed as box and whisker plot showing minimum to maximum values of n = 6-8 of two independent experiments. Statistical significance was determined using Mann-Whitney T-Test; **P< 0.01.

To study the role of CD200 in lung inflammation *in vivo*, we developed a Sprague-Dawley rat strain deficient in CD200 (KO) (**Supplementary Figure 1**). Given the high expression of CD200 receptor on AMs, we first characterized cells in BALs of both naïve WT and CD200 KO rats to determine whether CD200 deletion affected lung immune homeostasis. Surprisingly, BAL total cell numbers of CD200 KO rats were 44.6% lower than WT rats (**Figure 1A**), although they were both only comprised of AMs (**Figure 1A**). To determine whether change in AM numbers was associated with increased activation, AM expression of cell surface proteins was characterized, including SIRPα, CD11b, MHCII, and CD200R, but none were significantly different (**Figure 1B**). These data suggest that whilst depletion of CD200 reduces AM number, it does not influence AM activation status at homeostasis.

**Figure 2:**
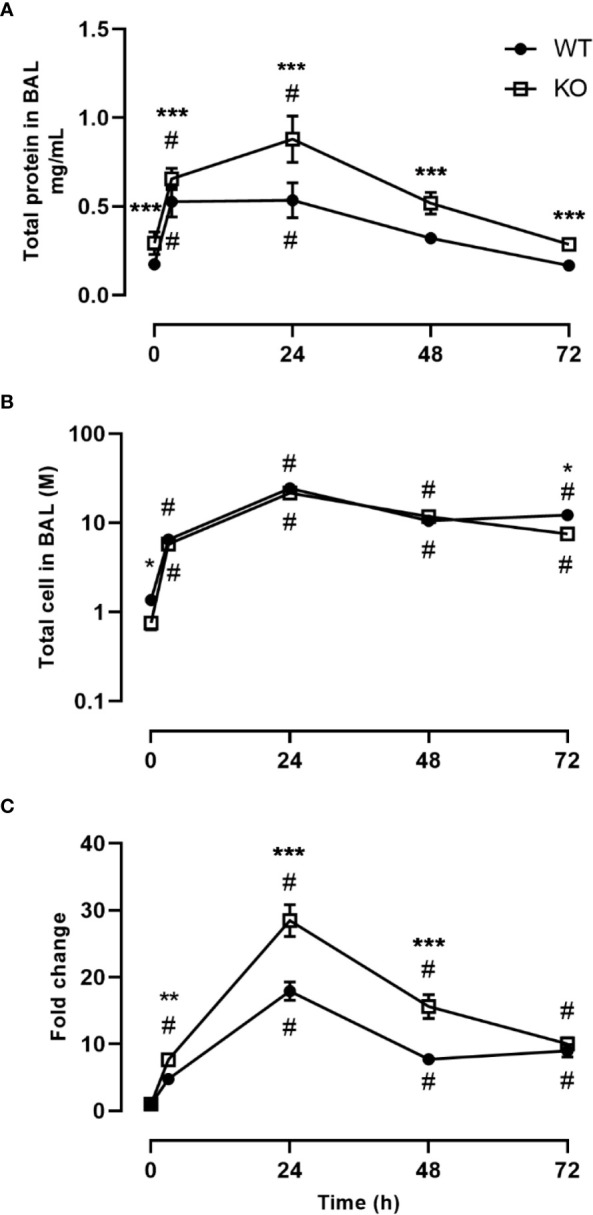
Increased lung damage and BAL inflammatory response to LPS exposure in CD200 KO. WT and CD200 KO rats were exposed to LPS intratracheally. Lung damage was assessed by **(A)** total protein in BAL, whereas inflammation was measured by **(B)** BAL total cell accumulation. **(C)** Total BAL cells were expressed as a fold change from baseline (time = 0h). Data are from individual rats, with n = 6-8 per group of two independent experiments. Statistical significance was determined using two-way ANOVA followed by Bonferroni’s multiple comparisons; * P < 0.05 vs WT; **P < 0.05 vs WT; ***P < 0.05 vs WT; ^#^P < 0.05 vs baseline.

**Figure 3:**
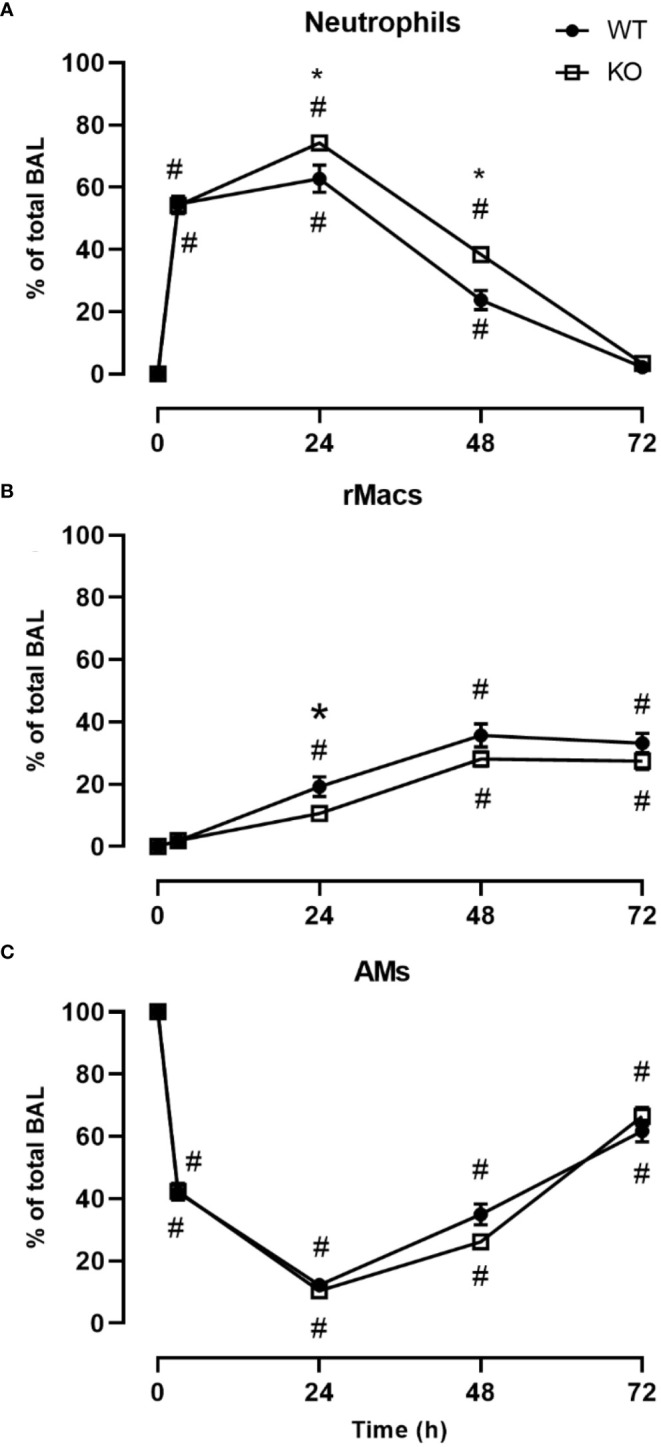
CD200 expression controls neutrophilic recruitment after LPS exposure. WT and CD200 KO rats were exposed to LPS intratracheally. BAL cell types were identified using flow cytometry, including, **(A)** Neutrophils, **(B)** recruited inflammatory macrophages (rMacs), and **(C)** alveolar macrophages (AMs). Data are from individual rats, with n = 6-8 per group of two independent experiments. Statistical significance was determined using two-way ANOVA followed by Bonferroni’s multiple comparisons; *P < 0.05 vs WT; ^#^P < 0.05 vs baseline.

We investigated the inflammatory response of CD200 KO rats in a model of ARDS using intratracheal administration of LPS. Lung injury was evaluated using total protein level in BALs, which is a hallmark biomarker of ARDS severity. At baseline, before LPS administration, protein level in BALs of CD200 KO rat was significantly higher than in WT rats and this difference increased over time following LPS exposure with a maximum at 24h (**Figure 2A**). Inflammatory response was characterized by measuring BAL immune cell recruitment after LPS administration. BAL cell accumulation peaked at 24h in WT and KO rats, and total cell numbers of CD200 KO rats were lower at baseline and after 72h post-LPS compared with WT (**Figure 2B**). Given the difference in cell numbers in naïve rats, the intensity of inflammatory response was expressed in fold increase from naïve rats and was significantly higher at 3, 24, and 48h in CD200 KO compared with WT rats (**Figure 2C**). When analyzing cellular composition of BAL, neutrophil recruitment was detected as soon as 3h post-LPS with a maximum accumulation at 24h and returned to baseline level at 72h (**Figure 3**). Neutrophil recruitment was similar in both rat strains at 3h but was significantly higher at 24 and 48 h in CD200 KO rats compared with WT rats. In contrast, macrophage recruitment (rMac) started at 24h and was still present at 72h (**Figure 3**). Interestingly, percentage of recruited macrophages (rMac) was transiently lower in CD200 KO rats at 24h. As expected, AM proportion declined after LPS exposure and was still significantly lower after 72h (**Figure 3**). This suggests that in this model of ARDS, acute inflammatory response is resolved at 72h post-LPS, although repair processes are not completed and back to homeostasis.

**Figure 4:**
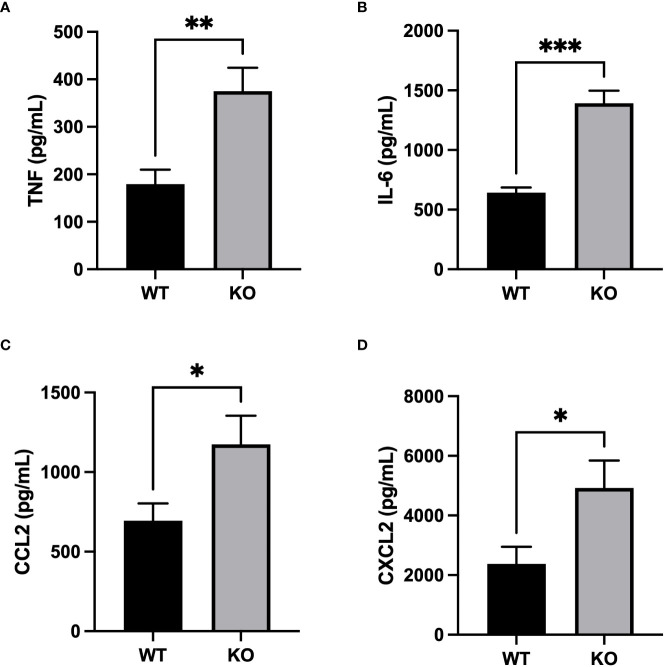
Increased production of inflammatory mediators in CD200 KO exposed to LPS. WT and CD200 KO rats were exposed to LPS intratracheally. Inflammatory mediators were measured in BAL fluid 3h post-LPS exposure by ELISA. BAL of CD200 KO rats had increased levels of **(A)** TNF, **(B)** IL-6, **(C)** CCL2, and **(D)** CXCL2. Data are from individual rats, with n = 6-8 per group of two independent experiments. Statistical significance was determined using Mann-Whitney T-Test; *P < 0.05; **P< 0.01; ***P< 0.001.

Inflammatory response severity was also measured by quantifying inflammatory mediators in BAL fluid. Concentrations of pro-inflammatory cytokines, TNF and IL-6, were significantly higher in BALs of CD200 KO rats compared with WT rats 3h after LPS administration (**Figures 4A, B**). Furthermore, levels of neutrophil chemokine, CXCL2, and macrophage chemokine, CCL2, were also higher in BALs of CD200 KO rats compared with WT rats (**Figures 4C, D**), suggesting a more robust inflammatory response in animals without CD200. Cytokine levels were also measured in BALs of naïve rats and at 24, 48, and 72 h after LPS exposure but concentration levels were below detection limit. These data show that in CD200 KO rats ARDS severity is acutely increased, although in both strains, inflammatory response is mostly resolved by 72h.

**Figure 5:**
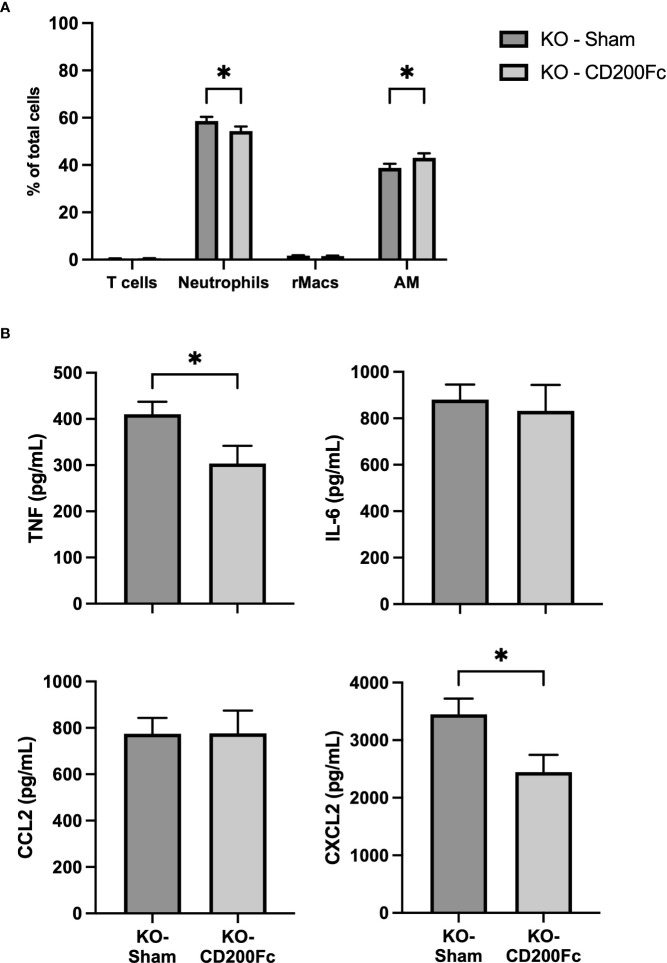
CD200Fc administration diminishes exacerbated acute inflammatory response in CD200 KO. CD200 KO rats were exposed to LPS intratracheally in combination with either recombinant IgG1 (sham) or CD200Fc, and BAL were performed 3h post-LPS. **(A)** BAL cell types were identified using flow cytometry, including T cells, Neutrophils, recruited inflammatory macrophages (rMacs), and alveolar macrophages (AMs). **(B)** Inflammatory mediators were measured by ELISA. CD200Fc treatment reduced levels of TNF and CXCL2, without altering IL-6 and CCL2 levels. Data are from individual rats, with n = 6-8 per group of two independent experiments. Statistical significance was determined using two-way ANOVA followed by Bonferroni’s multiple comparisons or Mann-Whitney T-Test; *P < 0.05.

To confirm the role of CD200 in reducing acute lung inflammation, CD200 KO rats were supplemented with CD200Fc at the same time than LPS administration and BALs were performed 3 h later. CD200Fc treatment did not alter BAL protein levels (data not shown) and total number of cells in BAL (respectively 5.6 ± 0.3 vs 5.4 ± 0.3 x 10^6^ cells in sham and CD200Fc). In contrast, proportion of neutrophils was significantly lower and proportion of AMs was higher in CD200Fc treated rats compared to sham treated rats (**Figure 5A**). Strikingly, only levels of TNF and CXCL2 were reduced in BALs of CD200 KO rats treated with CD200Fc (**Figure 5B**). IL-6 and CCL2 levels were not modulated by CD200Fc treatment (**Figure 5B**).

To evaluate which cell types might be the target of CD200Fc, we evaluated CD200R expression in BAL cell types at 3h post-LPS. Similarly to previous reports (12, 21), CD200R expression was higher in AM, with low level in rMACs and neutrophils (**Supplementary Figure 3**). This suggests that CD200 plays a role in reducing acute inflammatory response to LPS, likely by acting as an immune rheostat on AM immune response (10, 12, 25).

**Figure 6:**
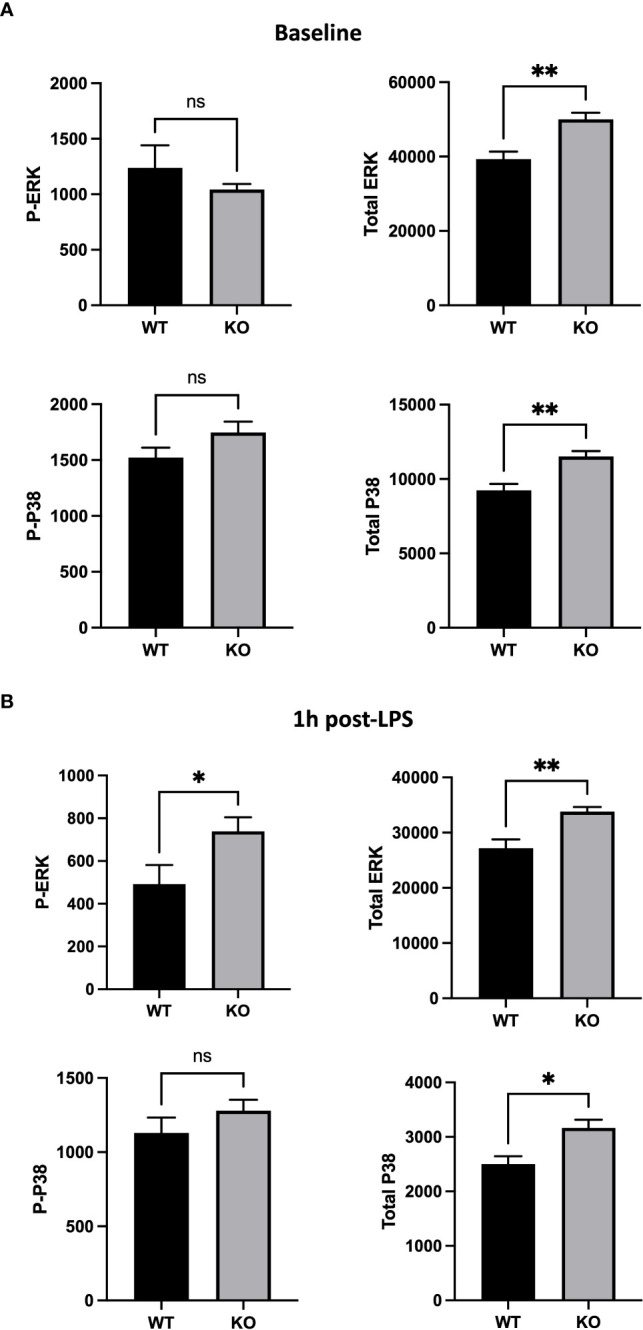
Alveolar macrophages (AMs) of CD200 KO rats have an higher expression of inflammatory MAP kinases. AMs of WT and KO rats were harvested at **(A)** baseline and **(B)** 1h post-LPS exposure. Expression of MAP kinases, ERK and P38, was assessed by flow cytometric analysis of delta mean fluorescence intensity. **(A)** At baseline, AMs of KO rats had increased levels of total ERK and P38, but similar levels of phospho (P-) ERK and P38. **(B)** 1h post-LPS, P-ERK and total ERK was increased in AMs of KO rats, as well as total P38. Data are from individual rats, with n = 6-8 per group of two independent experiments. Statistical significance was determined using Mann-Whitney T-Test; ns: not significant, *P < 0.05; **P< 0.01.

To further understand the role of CD200/CD200R pathway in AMs *in vivo*, we investigated expression of MAPKs that are involved in anti-inflammatory signaling of CD200R (26) and also in cellular response to LPS (27, 28). Using flow cytometry, we evaluated total and phosphorylated levels of 2 key molecules of MAPK pathway, ERK and P38, in AM of naïve and LPS stimulated rats. Although, there was no difference in phosphorylated ERK (P-ERK) and P38 (P-P38), there was significantly more total ERK and P38 in AMs of CD200 KO rats compared with WT (**Figure 6A**). 1h after LPS instillation *in vivo*, total level of ERK and P38 in CD200 KO rats remained elevated. Interestingly, after LPS, level of P-ERK, but not P-P38, was significantly higher in AMs from CD200 KO rats compared with WT rats (**Figure 6B**). These data suggest that CD200 plays an important role in lung homeostasis by limiting AM activation potential and after LPS exposure *in vivo*.

## Discussion

CD200/CD200R pathway is well known for its anti-inflammatory functions in many chronic inflammatory diseases (14). Moreover, absence of CD200 increases inflammatory response to viral infection by limiting viral replication but increasing lung injury because of uncontrolled inflammation (21). In this study, we showed that using an LPS-induced lung inflammation, absence of CD200 accentuated lung inflammatory response mainly *via* an increased activation of AMs.

Control of inflammatory response is complex, as inflammation is needed to eliminate pathogens but too much can cause injury. CD200/CD200R pathway represents one of the mechanisms involved in controlling inflammation, especially in the lung where its expression is high on both immune and epithelial cells (12, 20, 21). Its absence does not provoke an inflammatory status by itself but causes interesting immunologic alterations. Hoek et al. reported that absence of CD200 results in an increased number of macrophages in spleen suggesting that CD200 may be involved in regulation of macrophage lineage (17). In contrast, we found a dramatic reduction of AM number in BAL of CD200 KO rats. This difference may be explained, in part, by the fact that AMs are mainly derived from embryo yolk sac and fetal liver cells in contrast to macrophages in other tissues (29–31). This suggests that CD200 may be important for immune cell development from fetal cells but this would require additional investigation to confirm. Even though there were less AMs, absence of CD200 did not alter expression of AM surface markers such as SIRPα, CD11b, MHCII, and CD200R during homeostasis, as observed by Snelgrove et al. (21).

Strikingly, only part of the increased inflammatory response of CD200 KO to LPS was modulated by CD200Fc supplementation (**Figure 5**). Given that AMs express high levels of CD200R unlike other myeloid cells (21), regulation of TNF and CXCL2 levels by CD200Fc support that its mode-of-action is *via* binding to CD200R+ AMs which then leads to the reduction of neutrophil proportion. Opposingly, increased levels of IL-6 and CCL2 after LPS administration were unchanged by CD200Fc treatment, suggesting that production of these mediators by AMs is downstream of activation pathways not or weakly modulated by CD200R signaling cascade. Alternatively, AMs may not be the main producers of IL-6 and CCL2 mediators in our model, but rather epithelial cells (32–34) or other immune cells. Our data demonstrate that CD200/CD200R is likely acting as an immune rheostat in anti-inflammatory regulation in LPS-induced lung inflammation. Restraint control of lung inflammation by CD200Fc was also observed in an asthma model where CD200Fc reduced airway hyperresponsiveness and IL-13 level without modulating cell recruitment and eosinophil levels in BALs (22). Thus, CD200/CD200R pathway appears to have a selective role in controlling lung inflammation.

As a primary target of CD200/CD200R pathway, P-ERK is increased in AMs of CD200 KO rats following LPS, indicating higher activity of ERK pathway and thus increased secretion of pro-inflammatory cytokines measured in BALs. Interestingly, total ERK and P38 MAPK were significantly higher in CD200 KO rats compared with WT rats before and after LPS exposition. This could indicate a greater activation potential for MAPK pathways in CD200 KO rats. In addition of being a key regulator of inflammation, ERK signaling pathway arise an important player in cell proliferation, survival, and apoptosis (35). Hong et al. reported that activation of ERK over a certain threshold triggers cell growth arrest and death signaling (36) which could explain, in part, reduced number of AMs in BAL of CD200 KO rats. One the other hand, we are still puzzled by the higher level of total ERK/P38 in naïve AM. Given that unphosphorylated ERK/P38 have no known function, we can only speculate that a higher level of total ERK/P38 would allow a more rapid and robust phosphorylation cascade upon activation. Future projects will investigate this question in more details.

In conclusion, CD200/CD200R pathway shows selective regulation of acute lung inflammation and cannot completely abrogate complex inflammatory responses in a model of ARDS. However, addition of CD200 agonist in a multi-target therapy strategy could have beneficial impacts. Further investigation is needed to better understand the specificity of CD200/CD200R modulation of inflammation.

## Data availability statement

The raw data supporting the conclusions of this article will be made available by the authors, without undue reservation.

## Ethics statement

The animal study was reviewed and approved by Laval University Animal Care Committee in accordance with the guidelines of the Canadian Council on Animal Care.

## Author contributions

EB and J-FL-J designed and supervised the study. DP, KB, A-ML and J-FL-J performed the experiments. DP, EB and J-FL-J interpreted the data. DP, KB, EB and J-FL-J wrote and revised the manuscript. All authors contributed to the article and approved the submitted version.
