# Supplementary material for: Specificity of CD200/CD200R pathway in LPS-induced lung inflammation
Source: Front Immunol. 2022 Dec 15;13:1092126. doi: 10.3389/fimmu.2022.1092126 (PMC9797531; doi:10.3389/fimmu.2022.1092126)
Supplement: Supplementary file 1 [file DataSheet_1.pdf]

Sup Fig 1

A) B)

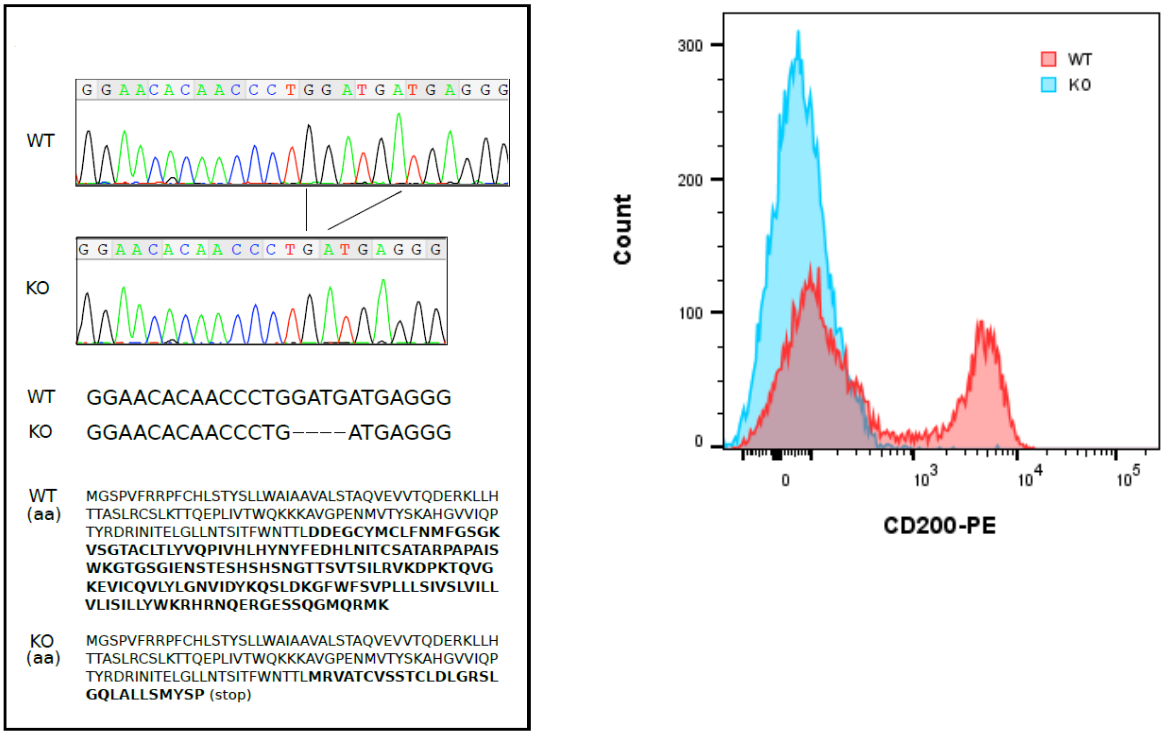

Sup Fig 1: Creation of CD200 knock-out rats.
Crl:CD(SD) Sprague Dawley rat ovocytes were injected with Cas9 mRNA and CD200-specific gRNA prior to implantation in pseudo-pregnant females.) A) Sequencing traces of F2 homozygous wild-type (WT) and CD200^{em1EB} knock-out (KO) rats. The translated sequence shows a 4bp deletion and the transcribed sequence shows a frameshift mutation (bold) with a premature stop codon in KO rats. B) Flow cytometry histogram of blood leukocytes stained with anti-rat CD200 antibodies shows absence of antibody binding in KO (blue) compared to WT (red).

Sup Fig 2

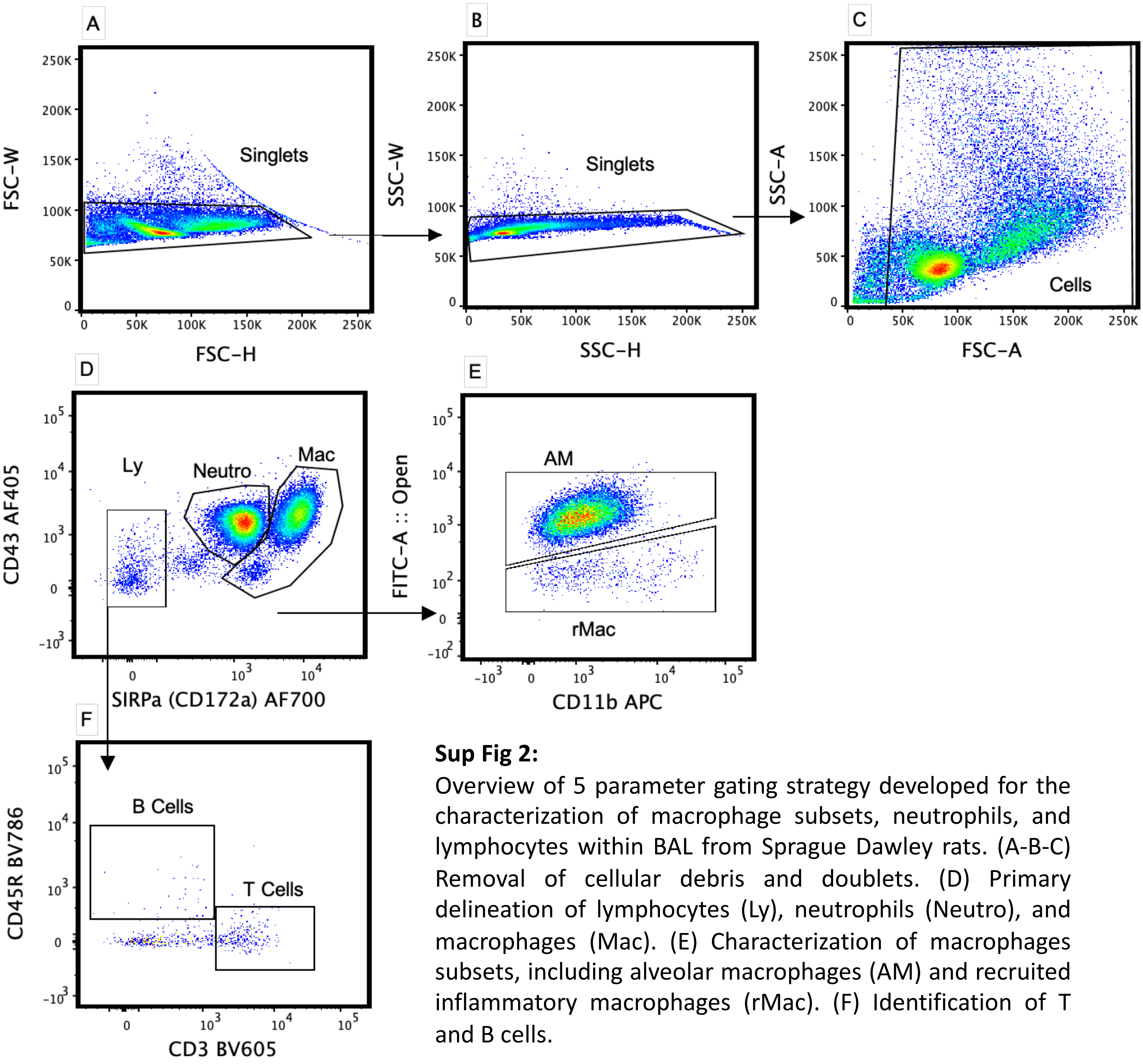

Sup Fig 2:

Overview of 5 parameter gating strategy developed for the characterization of macrophage subsets, neutrophils, and lymphocytes within BAL from Sprague Dawley rats. (A-B-C) Removal of cellular debris and doublets. (D) Primary delineation of lymphocytes (Ly), neutrophils (Neutro), and macrophages (Mac). (E) Characterization of macrophages subsets, including alveolar macrophages (AM) and recruited inflammatory macrophages (rMac). (F) Identification of T and B cells.

Sup Fig 3

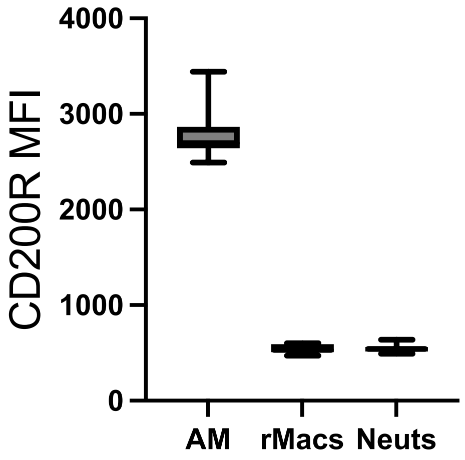

Sup Fig 3: CD200R expression in BAL

3h after LPS administration, CD200R expression was assed in BAL by flow cytometry. Expression level was measured using mean fluorescence intensity (MFI) and removing baseline level. AM: alveolar macrophages; rMACs: recruited inflammatory macrophages; Neuts: neutrophils.
